# WASp Is Essential for Effector-to-Memory conversion and for Maintenance of CD8^+^T Cell Memory

**DOI:** 10.3389/fimmu.2019.02262

**Published:** 2019-09-24

**Authors:** Qiao Liu, Liang Zhang, Zhou Shu, Tingting Yu, Lina Zhou, Wenxia Song, Xiaodong Zhao

**Affiliations:** ^1^Chongqing Key Laboratory of Child Infection and Immunity, Children's Hospital of Chongqing Medical University, Chongqing, China; ^2^Division of Immunology, Children's Hospital of Chongqing Medical University, Chongqing, China; ^3^Ministry of Education Key Laboratory of Child Development and Disorders, Chongqing, China; ^4^Department of Cell Biology and Molecular Genetics, University of Maryland, College Park, MD, United States

**Keywords:** Wiskott-Aldrich syndrome, CD8 T cells, memory, kinetics, recall response

## Abstract

Wiskott-Aldrich syndrome (WAS) is a rare X-linked primary immunodeficiency characterized by recurrent infections, micro thrombocytopenia, eczema, and a high incidence of autoimmunity and malignancy. A defect in the T cell compartment is thought to be a major cause of immunodeficiency in patients with WAS; However, whether the antigen specific T memory cell is altered has not been extensively studied. Here, we examined the expansion/contraction kinetics of CD8^+^ memory T cells and their maintenance in WASp^−/−^ mice. The results showed that WAS protein (WASp) is not required for differentiation of CD8^+^ effector T cells; however, CD8^+^ T cells from WASp^−/−^ mice were hyperactive, resulting in increased cytokine production. The number of CD8^+^ T memory cells decreased as mice aged, and CD8^+^ T cell recall responses and protective immunity were impaired. WASp-deficient CD8^+^ T cells in bone marrow chimeric mice underwent clonal expansion, but the resulting effector cells failed to survive and differentiate into CD8^+^ memory T cells. Taken together, these findings indicate that WASp plays an intrinsic role in differentiation of CD8^+^ memory T cells.

## Introduction

Wiskott-Aldrich syndrome (WAS) is a rare X-linked primary immunodeficiency characterized by recurrent infections, micro thrombocytopenia, eczema, and a high incidence of autoimmunity and malignancy (1). The disease is caused by mutations in the WAS gene on the X-chromosome, which encodes the Wiskott-Aldrich syndrome protein (WASp) (2). WASp belongs to a family of proteins that participates in organization of actin polymerization, primarily through activation of the actin-related protein (Arp2/Arp3) complex.

Compromised cellular adaptive immunity is a hallmark of classical WAS. T cell lymphopenia, which is common in WAS patients, is caused by abnormal T cell development and thymic output, as well as increased apoptosis (3–7). Naïve T cells from WAS patients consistently fail to upregulate the activation markers CD69 and CD25, proliferate, or secrete interleukin (IL)-2, interferon (IFN)-γ, and tumor necrosis factor in response to TCR/CD28-mediated activation (8–11). In addition, lytic granule polarization in CD8^+^ T cells is incomplete (12). These data provide evidence as to why WAS patients are prone to infection. Intriguingly, the incidence of infection, especially viral infection, increases markedly with age (1). Memory CD8^+^ T cells play a critical protective role against the virus infection (13); therefore, we hypothesized that T cell memory is compromised in WAS patients.

After virus infection, CD8 T cells underwent three critical phases: the activation and expansion phase [prior to D8 post-infection (PI)], the effector-to-memory transition phase (D9–D30 PI), and the maintenance phase (D30 PI and later) (13, 14). T cell activation by dendritic cells (DCs) involves integration of multiple signals induced by antigens, costimulatory molecules, cytokines, and chemokines. Together, these signals regulate differentiation of effector and memory T cells. WASp plays an important role in formation of the phagocytic cup (15) and podosomes (16) in macrophages and dendritic cells. DC homing from peripheral tissues to T cell zones in the secondary lymphoid tissues of WASp knockout (WASp^−/−^) mice is kinetically and quantitatively impaired (17, 18). As naïve T cells are programmed to recognize antigen only in the T cell zones of secondary lymphoid tissues, the defect in WASp^−/−^ T cells with respect to homing and interaction with impaired DCs may markedly reduce generation of T memory cells. In addition, WASp may play a critical role in migration of memory T cells, which is a key characteristic of this subclass and is essential for their immunosurveillance function.

Although T cell defects are a major cause of immunodeficiency in patients with WAS, it is not completely clear whether antigen-specific T cell memory is altered. Previous studies suggest that CD8^+^ T cell memory is impaired in mice bearing WAS mutations (19, 20). However, the stage of CD8^+^ T memory cell differentiation in which WASp is involved is unclear; nor is it clear whether the function of CD8^+^ memory T cells in WASp^−/−^ mice is normal, or whether the roles of WASp are intrinsic to CD8^+^ T cells.

The mechanisms underlying the generation and rapid recall ability of CD8^+^ memory T cells are unclear. Here, we examined the role of WASp during expansion, contraction, maintenance, and recall responses of memory CD8^+^T cells in response to lymphocytic choriomeningitis virus (LCMV) infection. We found that WASp deficiency increased expansion of CD8^+^ T cells in response to infection but decreased the differentiation of central memory T cells (TCM) from primed CD8^+^ T cells during the contraction phase, resulting in a reduced pool of memory CD8^+^ T cells. Furthermore, the data indicate that WASp expressed by CD8^+^ T cells is responsible directly for recall responses and protection from re-infection. To the best of our knowledge, this is the first study to show an intrinsic role for WASp in the contraction and maintenance of CD8^+^ T cells in response to viral infection. The results highlight the importance of WASp in facilitating survival of primed CD8^+^ T cells during the maintenance phase.

## Materials and Methods

### Mice, Viruses, and Infection

WASp^−/−^ mice (C57BL/6) and their littermates were obtained from Jackson Laboratories and bred in-house in single ventilated cages. LCMV-Armstrong and a recombinant strain of L. monocytogenes (modified to secrete a segment of LCMV-GP (Lm-GP33)) were kind gifts from Professor Li lin Ye [Third Military Medical University]. Mice, aged 6–8 weeks, were infected intraperitoneally (i.p) with LCMV-Armstrong (2 × 10^5^ PFU) or intravenously with Lm-GP33 (1 × 10^5^ PFU). Bone marrow (BM) chimeras were infected after 8 weeks of reconstitution. Mice infected with LCMV and Lm-GP33 were housed in accordance with the institutional biosafety regulations of Chong Qing Medical University. All animal experiments were conducted in accordance with the guidelines of the Institutional Animal Care and Use Committees of Chong Qing Medical University.

### Flow Cytometry Analysis

Cell surface staining was performed in PBS containing 2% FBS. To examine production of intracellular cytokines, splenocytes were first stimulated for 5 h at 37°C with GP33-41 peptides (0.2 μg/ml) and brefeldin A. Following surface staining, intracellular cytokine staining was performed using a Cytofix/Cytoperm Fixation/Permeabilization Kit (554714, BD Biosciences), according to the manufacturer's instructions. To detect degranulation, splenocytes were stimulated for 5 h in the presence of the indicated peptide (0.2 μg/ml), brefeldin A, and anti-CD107a. Major histocompatibility complex class I peptide tetramers derived from H-2Db complexed with LCMV GP33-41 were purchased from MBL (Japan). Cells were analyzed using a FACSCanto II (BD Biosciences) cytometer and data were analyzed using FlowJo software (Treestar).

### Quantification of LCMV and Lm-GP33 Titers

A qRT-PCR assay was used to measure the LCMV load in serum and liver, as described previously (21). To measure the bacterial load in internal organs, the liver and spleen were removed aseptically and homogenized. Serial dilutions were prepared and plated on Brian Heart Infusion (BHI) agar. After 24 h, the numbers of bacterial colonies were counted to measure the colony-forming unit(CFU)and the results presented as CFU/g organ tissue.

### Adoptive Transfer

CD8^+^ T cells were purified by negative-selection on magnetic beads (MACS; stem cell). The purity was >95%. To examine recall responses, an equal number of CD8^+^GP33^+^ T cells from wild-type (WT) and WASp^−/−^ (CD45.2) mice were adoptively transferred (intravenously) into naïve CD45.1 recipient mice. One day later, mice were challenged intravenously with 1 × 10^5^ PFU of Lm-GP33. Spleens were harvested 5 days later and stained with GP33-41 tetramer and antibodies specific for anti-CD8, anti-CD45.2, anti-CD44, and anti-CD45.1. To calculate the -fold expansion of transferred virus-specific memory CD8^+^ T cells, the total number of virus-specific memory CD8^+^ T cells in the spleen of each mouse (at 5 days post-challenge) was calculated and divided by the number of virus-specific CD8^+^ T cells initially transferred into the recipient (before challenge).

#### BM Chimera

BM cells from C57BL/6J (CD45.2) or WASp^−/−^ (CD45.2) mice, and BM cells from C57BL/6J (CD45.1) mice, were mixed and adoptively transferred (intravenously at a 1:1 ratio) into lethally irradiated (two doses, each of 450 rad) WT C57BL/6J (CD45.1) mice. A total of 5 million BM cells per mouse were transferred. Recipient mice were fed antibiotics for 2 weeks and allowed to reconstitute for 8 weeks prior to infection.

### Statistical Analysis

Data analysis was performed using Graph Pad Prism 7.0a (Graph pad Software, USA). Flow cytometry data were analyzed using FlowJo 10.3 (Tree Star Inc., USA). A *P*-value of 0.05 was deemed significant.

## Results

### Efficient Clonal Expansion and Effector Differentiation of WASp-Deficient CD8^+^ T Cells

To investigate the role of WASp in regulating Ag-driven generation, contraction, and maintenance of memory CD8^+^ T cells, we used WASp^−/−^ mice with an acute LCMV infection. At first, we studied uninfected mice (6–8 weeks old) and found a lower frequency and absolute number of CD8^+^ T cells in the spleen of WASp^−/−^ mice than in WT mice (Supplemental Figure 1A). The percentage and absolute number of the naïve CD8^+^ T cells (CD44^lo^CD62L^hi^) in the spleen of WASp^−/−^ mice was consistently low (Supplemental Figure 1B). However, the absolute number of TCMs (CD44^hi^CD62L^hi^) and effector memory T cells (TEMs, CD44^hi^CD62L^lo^) in WASp^−/−^ mice was comparable with that in uninfected WT mice (Supplemental Figures 1C,D).

**Figure 1:**
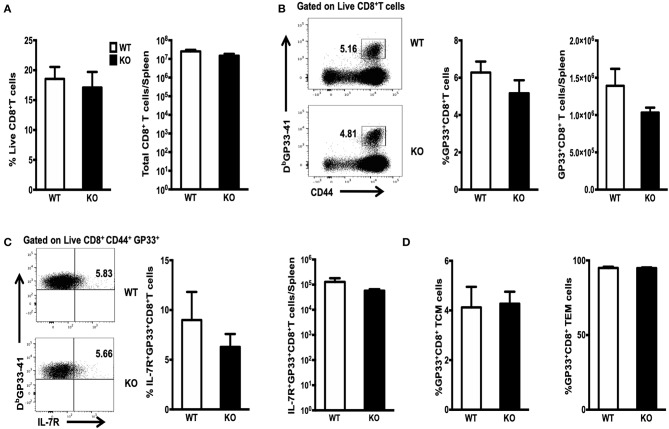
Efficient clonal expansion and effector differentiation of WASp-deficient CD8^+^ T cells. WT and WASp^−/−^ mice were infected with LCMV and the number of GP33-specific CD8 T cells in the spleen on 8 days PI was quantified by staining with an anti-CD8 antibody and D^b^/GP33 tetramers. Bar graphs in **(A)** illustrate the percentage (left panel) and absolute number of CD8^+^ T cells (right panel) in the spleen on Day 8 PI. **(B)** Flow cytometry analysis of spleenocytes from WT and WASp^−/−^ mice; numbers indicate the percentage of GP33-specific cells within the CD8^+^ T cell population (left panel) and the histogram show the percentage of GP33-specific T cells within CD8^+^T cells (middle panel) and the absolute number of GP33-specific CD8^+^ T cells (right panel) in the spleen on Day 8 PI. **(C)** Representative data showing expression of IL-7R on GP33 specific CD8^+^T cells (left panel); Percentages of IL-7R^+^ cells within the GP33-specific CD8^+^ T cell population (middle panel) and the absolute number of IL-7R^+^ GP33-specific CD8^+^ T cells (right panel) in spleen on Day 8 PI. **(D)** The proportion of TCM (left panel) and TEM (right panel) within the GP33-specific CD8^+^ T cell population was determined by measuring expression of CD62L. Data are expressed as the mean ± SEM and are representative of at least two independent experiments, each with 5 mice per group. Data were analyzed using an unpaired *t*-test.

The population of LCMV-specific effector CD8^+^ T cells normally peaks on Day 8 PI. Only a small proportion of these CD8^+^ T cells survives and differentiates into memory T cells. This subset can be identified by expression of the IL-7 receptor (IL-7R) (22–24). We infected WT or WASp^−/−^ mice with LCMV and analyzed antigen-specific CD8^+^ T cell responses on Day 8. Strikingly, both the percentage and absolute number of CD8^+^T cells in the spleen of WASp^−/−^ mice remained the same as those in WT mice (Figure 1A). The percentage and total number of CD8^+^ T cells specific for immunodominant epitope GP33-41 (GP33) in WASp^−/−^ mice was comparable with those in WT mice (Figure 1B). As expected, only a small fraction of GP33-specific CD8^+^ T cells in WT and WASp^−/−^ mice were IL-7R-positive; in addition, the percentage and total number of IL-7R^+^ GP33^+^CD8^+^ T cells in WASp^−/−^ mice was comparable with those in WT mice (Figure 1C). Next, we divided GP33-specific CD8^+^ T cells into TCMs and TEMs according to expression of CD62L; however, we found no significant difference between WT and WASp^−/−^ mice (Figure 1D). There was also no significant difference in expression of BCL-2, Ki67, or T-bet between WASp^−/−^ GP33-specific CD8^+^ T cells and WT GP33-specific CD8^+^ T cells (Supplemental Figure 2A). These findings suggest that WASp deficiency does not have a significant effect on expansion of LCMV-specific CD8^+^ T cells *in vivo* during acute viral infection.

**Figure 2:**
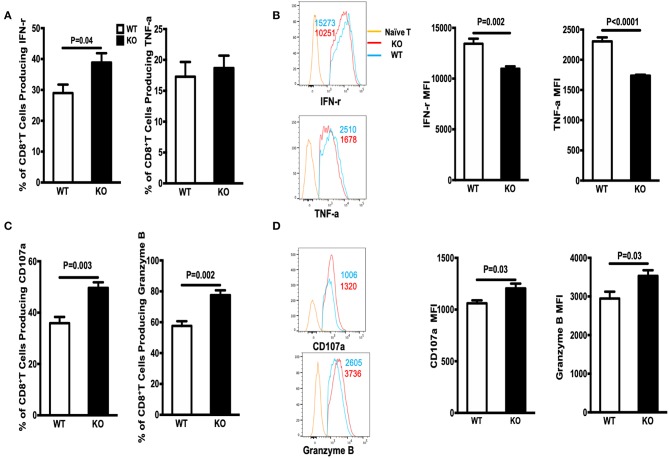
Cytokine production by WASp^−/−^ mice is altered during the expansion stage. Cytokine production was analyzed by flow cytometry after re-stimulation of *in vivo*-primed (Day 8) virus-specific T cells for 5 h. **(A)** The percentages of IFN-γ^+^ cells (left panel) or TNF-α^+^ cells (right panel) within the CD8^+^ T cell population in the spleen. **(B)** Representative data of IFN-γ expression (left upper panel, gated on Live CD8^+^CD44^+^ IFN-γ^+^ cells) and TNF-α expression (left lower panel, gated on Live CD8^+^CD44^+^ TNF-α^+^ cells); the numbers in the histograms represent the MFI for the indicated protein, naïve T cell were used as a negative control. **(C)** The percentages of CD107α- and Granzyme B-producing cells within the CD8^+^ T cell population. **(D)** representative data of CD107α expression (left upper panel, gated on Live CD8^+^CD44^+^ CD107α ^+^ cells) and Granzyme B expression by Granzyme B^+^CD8^+^ T cells (left lower panel, gated on Live CD8^+^CD44^+^ Granzyme B^+^ cells), the numbers in the histograms represent the MFI for the indicated protein, naive T cell were used as a negative control. Data are expressed as the mean ± SEM, each with 5 mice per group. Data were analyzed using an unpaired *t*-test.

Next, we examined the ability of GP33-specific CD8^+^ effector T cells from WT and WASp^−/−^ mice (on Day 8 PI) to produce cytokines in response to Ag stimulation *in vitro*. Spleenocytes were stimulated for 5 h at 37°C with GP33-41 peptides (0.2 μg/ml) and brefeldin A. CD8^+^ effector T cells from both WT and WASp^−/−^ mice produced readily detectable levels of IFN-γ, TNF-α, CD107α, and Granzyme B upon stimulation with the cognate antigenic peptide. The percentage of IFN-γ-producing CD8^+^ T cells in WASp^−/−^ mice was higher than that in WT mice, whereas the frequency of TNF-α-positive CD8^+^ T cells in WT and WASp^−/−^ mice was not significantly different (Figure 2A). However, the amount of IFN-γ and TNF-α produced (based on measurement of mean fluorescence intensity [MFI]) by WASp^−/−^ effector CD8^+^ T cells was significantly lower than that produced by WT effector CD8^+^ T cells (Figure 2B). As indices for the cytolytic function of effector CD8^+^ T cells, we measured expression of CD107α and Granzyme B in GP33-specific CD8^+^ T cells *ex vivo*. Strikingly, both the percentage of CD107α- and Granzyme B-positive CD8^+^ T cells and the amount of CD107α and Granzyme B produced by CD8^+^ T cells were significantly higher in WASp^−/−^ mice than in WT mice (Figures 2C,D). This infers that GP33-specific CD8^+^ T cells recovered from WASp^−/−^ mice during the expansion stage were hyperactive. Irrespective of the higher amount of CD107α and Granzyme B produced by CD8^+^T cells in WASp^−/−^ mice, we found reduced viral clearance from the liver of WASp^−/−^ mice on D8 PI (Supplemental Figure 2B).

### Effector CD8^+^ T Cell Contraction and Early Memory Cell Formation Are Partially Impaired in WASp^−/−^ Mice

**Figure 3:**
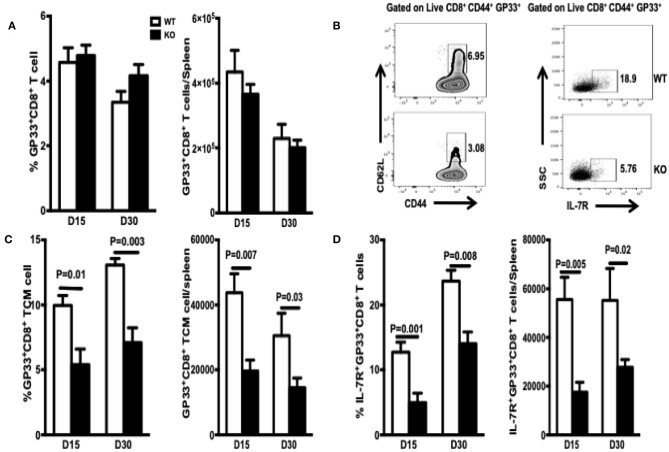
TCM and MEPC numbers in WASp^−/−^ mice fall during the contraction and early memory stages. TCM are defined as CD44^high^ CD62L^high^. MEPC are defined as IL-7R^high^. TCM and MEPC were gated on CD44^+^GP33^+^ CD8^+^ T cells. **(A)** Percentages of GP33-specific cells within the CD8^+^ T cell population (left panel) and absolute number of GP33-specific CD8^+^T cells in the spleen (right panel). **(B)** Left panel: representative data for TCM on D15; right panel, representative data for MEPC on D15. **(C)** Percentages of TCMs within the GP33-specific CD8^+^ T cell population and absolute number of GP33-specific TCMs in the spleen. **(D)** Percentage of MEPCs within the GP33-specific CD8^+^ T cell population and absolute number of GP33-specific MEPC in the spleen. Data are expressed as the mean ± SEM (*n* = 5 mice per group) and were analyzed using an unpaired *t*-test.

Differentiation of naïve T cells into memory CD8^+^ T cells occurs via two keys steps. The first is conversion of naïve T cells to effector cells, which execute the cytotoxic function. The second is differentiation of effector cells into functional CD8^+^ memory T cells (25). Generation of functional memory T cells requires successful passage through these two checkpoints. During the 2–4 weeks PI, CD8^+^ effector T cells undergo effector-to-memory transition, which is characterized by a functional change from robust killing to resting; however, there is a poised state that enables these cells to expand clonally and develop effector functions rapidly upon re-exposure to Ag. First, we assessed whether WASp affects contraction of CD8^+^ effector T cells and their differentiation into early CD8^+^ memory T cells. The frequency and total number of GP33-specific CD8^+^ T cells in WASp^−/−^ mice recovered on Days 15 and 30 PI were similar to those in WT mice (Figure 3A). Thus, WASp deficiency seemed to have no effect on contraction of GP33-specific CD8^+^ T cells. However, because effector-to-memory transition leads to enrichment of TCMs, we examined the effect of WASp deficiency on the proportion of TEMs and TCMs. We found a marked reduction in the percentage and absolute number of GP33-specific TCMs in WASp^−/−^ mice (Figures 3B,C). Regardless of CD62L expression, there was a significantly higher proportion and absolute number of IL-7R-positive GP33-specific CD8^+^ T cells in WT mice than in WASp^−/−^ mice (Figures 3B,D), suggesting that more memory cells are generated in WT mice than in WASp^−/−^ mice at the maintenance stage. The reduction in TCM formation and IL-7R expression by WASp^−/−^ GP33-specific CD8^+^ T cells suggests disruption of CD8^+^ T cell effector contraction and memory transition. Collectively, the data suggest that WASp plays a crucial role in effector-to-memory transition during an acute viral infection.

**Figure 4:**
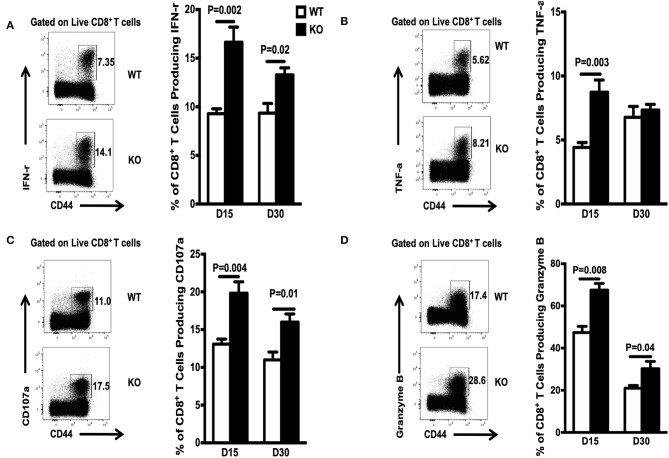
Increased accumulation of cytokine-producing CD8^+^T cells during the contraction and early memory stages in WASp^−/−^ mice. On D15 and D30 after LCMV infection, spleenocytes were stimulated with GP33-41 peptide *in vitro* and Ag-induced cytokine production was measured by intracellular staining. Representative data showing cytokine production on D30. **(A)** Percentage of IFN-γ-producing cells within the CD8^+^ T cell population. **(B)** Percentage of TNF-α producing cells within the CD8^+^ T cell population. **(C)** Percentage of CD107-α producing cells within the CD8^+^ T cell population. **(D)** Percentage of Granzyme B-producing cells within the CD8^+^ T cell population. Data are expressed as the mean ± SEM (*n* = 5 mice per group) and were analyzed using an unpaired *t*-test.

To mature functionally, CD8^+^ memory T cells need to acquire the ability to produce multiple cytokines, including IFN-γ, TNF-α, and CD107α. To assess the effects of WASp deficiency on functional maturation of CD8^+^ T cells, we measured *in vitro* cytokine production by CD8^+^ T cells on Days 15 and 30 PI. The percentage of cytokine-producing CD8^+^ T cells from WASp^−/−^ mice was significantly higher than that from WT mice (Figures 4A–D). However, the amount of IFN-γ, TNF-α, CD107α, and Granzyme B produced (based on MFI) by WASp^−/−^ effector CD8^+^ T cells was comparable with that produced by WT effector CD8^+^ T cells (data not shown). These results, together with reduced expression of IL-7R, suggest that, unlike in WT mice, GP33-specific effector CD8^+^ T cells in WASp^−/−^ mice have not reached a quiescent state. However, the LCMV load in the liver and the serum of both WT and WASp^−/−^ mice was undetectable by Day 30 PI (data not shown). Taken together, these data suggest that WASp plays a non-redundant role in cytokine production in effector CD8^+^ T cells and functional maturation of CD8^+^ memory T cells.

### WASp Deficiency Limits the Size of the CD8^+^ Memory T Cell Compartment and Promotes CD8^+^ T Cell Exhaustion

**Figure 5:**
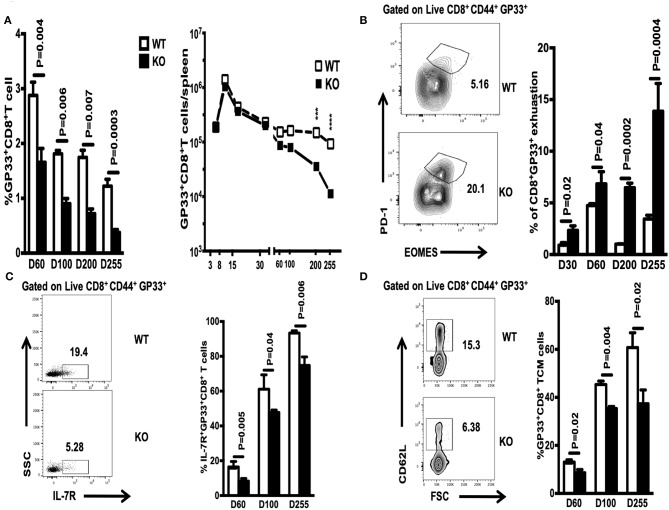
WASp deficiency limits the size of the CD8^+^ memory T cell compartment and promotes CD8^+^ T cell exhaustion. On the indicated days PI, WT and WASp^−/−^ mice were scarified and the percentage and absolute number of GP33-specific CD8^+^ T cells in the spleen were enumerated by Tetramer staining. **(A)** Percentage and absolute number of GP33-specific CD8^+^ T cells on the indicated days. **(B)** Left panel, representative data for exhausted T cells on D255; right panel, percentage of exhausted CD8^+^ T cells within the GP33-specific cell population on the indicated days. **(C)** Left panel, representative data showing IL-7R^+^ GP33^+^ T cells on D60; right panel, percentage of IL-7R^+^ GP33-specific CD8^+^ T cells on the indicated days. **(D)** Left panel, representative data showing TCMs on D60; right panel, percentage of TCMs on the indicated days. Data are expressed as the mean ± SEMs (*n* = 3 to 8 mice) (WT littermate mice: D30, *n* = 4; D60, *n* = 7; D100, *n* = 7; D200, *n* = 3; D255, *n* = 5; WASp^−/−^ mice: D30, *n* = 4; D60, *n* = 8; D100, *n* = 7; D200, *n* = 3; D255, *n*= 5). Data were analyzed using an unpaired *t*-test.

**Figure 6:**
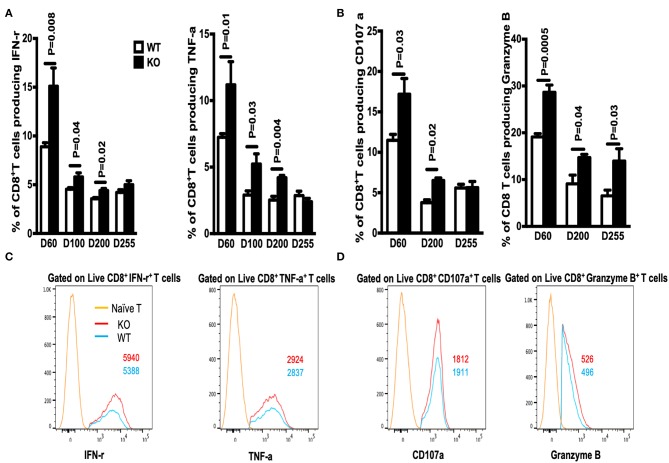
The percentage of cytokine-producing CD8^+^ T cells in WASp^−/−^ mice increases during the maintenance stage. On the indicated days after LCMV infection, spleenocytes were stimulated with GP33-41 peptide *in vitro* and Ag-induced cytokine production was measured by intracellular staining. **(A)** Percentages of IFN-γ^+^ (left panel) or TNF-α^+^ cells (right panel) within the CD8^+^ T cell population. **(B)** Percentages of CD107a^+^ (left panel) or Granzyme B^+^ cells (right panel) within the CD8^+^ T cell population. **(C)** Representative data showing expression of IFN-γ (left panel, gated on Live CD8^+^CD44^+^ IFN-γ^+^ cells), and expression of TNF-α (right panel, gated on Live CD8^+^CD44^+^ TNF-α^+^cells). **(D)** Representative data showing CD107α expression (left panel, gated on Live CD8^+^CD44^+^ CD107α^+^ cells), and Granzyme B expression (right panel, gated on Live CD8^+^CD44^+^ Granzyme B^+^ cells). Representative data showing cytokine production on D60; naïve T cells were used as a negative control. The numbers in the histograms represent the MFI for the indicated protein. Data are expressed as the mean ± SEM (*n* = 3–8 mice) (WT littermate mice: D30, *n* = 4; D60, *n* = 7; D100, *n* = 7; D200, *n* = 3; D255, *n* = 5; WASp^−/−^ mice: D30, *n* = 4; D60, *n* = 8; D100, *n* = 7; D200, *n* = 3; D255, *n* = 5). Data were analyzed using an unpaired *t*-test.

The number of memory CD8^+^ T cells during a T cell response is controlled by expansion, contraction, and maintenance phases (14). Here, we asked whether WASp deficiency influences the proportion and absolute number of GP33-specific CD8^+^ memory T cells during the maintenance stage. We found that the percentage of GP33-specific CD8^+^ T cells in the spleen of WASp^−/−^ mice after Day 60 PI was lower than that in WT mice, and that this low percentage was maintained for up to 255 days PI (Figure 5A, left panel); however, because the spleens of WASp^−/−^ mice are larger than those of WT mice, the same as previous described (19), the absolute number of GP33-specific CD8^+^T cells fell only up until D200 and D255 (Figure 5A, right panel). Meanwhile, the percentage of exhausted T cells, as indicated by EOMES and PD-1 staining, within the LCMV GP33-specific CD8^+^ T cell population in WASp^−/−^ mice increased markedly compared with that in WT mice (Figure 5B). However, the frequency of IL-7R^+^ GP33-specific T cells, and that of TCMs within the GP33-specific T cell population, was consistently lower in WASp^−/−^ mice than in WT mice (Figures 5C,D). WASp^−/−^ mice showed a marked increase in the percentage of IFN-γ-, TNF-α-, CD107α-, and Granzyme B-producing CD8^+^T cells upon stimulation with a cognate antigenic peptide on Day 60 PI and at later time points (Figures 6A,B), although the MFI of these cytokine was comparable (Figures 6C,D). These data strongly imply that WASp plays an important role in maintaining the magnitude and function of CD8^+^ T cell memory in the spleen following acute viral infection.

### WASp Deficiency Increases Apoptosis of Antigen-Specific CD8^+^ T Cells via the Extrinsic Pathway

**Figure 7:**
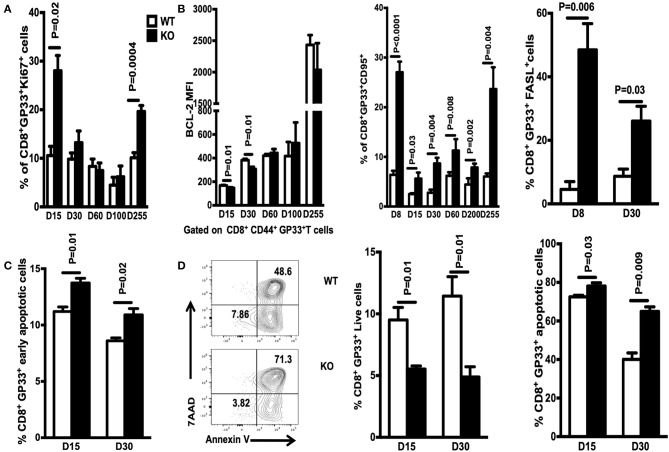
GP33-specific CD8 T cells from WASp^−/−^ mice display increased apoptosis. Spleenocytes were stained for CD8, CD44, Db/GP33 tetramer, FAS, and FASL. Ki67 and BCL-2 were detected by intracellular staining **(A)** Percentage of Ki67^+^ cells within the GP33-specific CD8^+^T cell population in the spleen on the indicated days. **(B)** Left panel, expression of BCL-2 by GP33-specific CD8^+^ T cells on indicated days; middle and right panels, percentage of FAS- and FASL-positive cells within the GP33-specific cell population in the spleen on the indicated days. **(C)** Apoptosis was determined by staining with antibodies specific for CD8 and CD44, the Db/GP33 tetramer, and with 7-AAD, and Annexin V immediately after isolation on D15 and D30 PI. **(D)** Left panel, representative data showing apoptosis on D30 after culture of spleenocytes with GP33-41 peptide for 24 h; middle and right panels, percentage of live or apoptotic GP33-specific T cells. Data are expressed as the mean ± SEM (*n* = 3–8 mice), Data were analyzed using an unpaired *t*-test. For **(A,B)** WT littermate mice: D15, *n* = 5; D30, *n* = 4; D60, *n* = 7; D100, *n* = 7; D200, *n* = 3; D255, *n* = 5; WASp^−/−^ mice: D15, *n* = 5; D30, *n* = 4; D60, *n* = 8; D100, *n* = 7; D200, *n* = 3; D255, *n* = 5. For **(C,D)** D15, *n* = 3; D30, *n* = 4.

Next, we asked whether the reduction in the number of antigen-specific CD8^+^ T cells observed in WASp^−/−^ mice is due to an abnormal thymic output. WT and WASp^−/−^ mice had comparable numbers of total thymocytes and CD8^+^ single-positive subsets, which showed similar expression of thymocyte-maturation markers such as CD62L and CD69 (Supplemental Figures 3A,B). This indicates that the thymic output of CD8^+^ T cells is relatively normal in WASp^−/−^ mice. The other possibility was decreased proliferation of WASp^−/−^ CD8^+^ T cells. However, proliferation of GP33-specific CD8^+^T cells (as indicated by expression of Ki 67) was even higher in WASp^−/−^ mice (Figure 7A). Next, we asked whether increased apoptosis might play a role in the reduction of antigen-specific CD8^+^ T cell numbers in WASp^−/−^ mice. T cell apoptosis is mediated by two pathways: the intrinsic pathway (controlled by the Bcl-2 family) and the extrinsic pathway (induced by signals from death receptors such as Fas) (26). To test our hypothesis, we compared expression of BCL-2, FasL, and Fas in WASp^−/−^ and WT GP33-specific CD8^+^ T cells. GP33-specific CD8^+^ T cells from WASp^−/−^ mice expressed BCL-2 at levels that were slightly lower or comparable with those of their WT counterparts (Figure 7B, left panel). However, we consistently detected a higher percentage of CD95- (Fas) and CD178 (FasL)-positive GP33-specific CD8^+^ T cells in WASp^−/−^ mice than in WT mice at different time points PI (Figure 7B, middle and right panels). Next, we quantified apoptosis in GP33-specific CD8^+^ T cells on Days 15 and 30 PI. We found that early apoptosis (AnnexinV^+^7AAD^−^) of GP33-specific CD8^+^ T cells in WASp^−/−^ mice was higher than that in WT mice (Figure 7C), while the proportion of apoptotic cells (AnnexinV^+^7AAD^+^) was comparable (data not shown). After stimulation with GP 33-41 peptide for 24 h, the percentage of live (AnnexinV^−^7AAD^−^) GP33-specific CD8 T cells was significantly higher, and the proportion of apoptotic cells consistently lower, in WT mice than in WASp^−/−^ mice (Figure 7D). This suggests a more activated state, along with up-regulation of the Fas pathway in WASp^−/−^ GP33-specific CD8^+^ T cells.

### WASp^−/−^ Mice Mount Impaired Secondary CD8^+^ T Cell Responses

**Figure 8:**
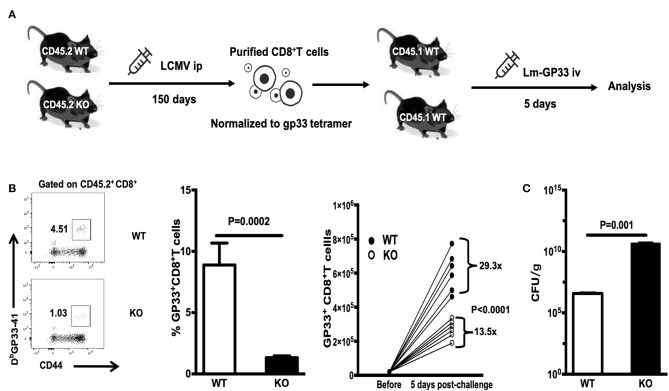
WASp^−/−^ mice mount impaired secondary CD8^+^ T cell responses. WT and WASp^−/−^ mice were sacrificed on D150 PI. Equal numbers of GP33^+^CD8^+^ T cells were adoptively transferred to naïve congenic recipients, which were challenged on the next day with Lm-GP33. At 5 days post-challenge, the -fold increase in the number of transferred GP33-specific CD8^+^ T cells was determined. **(A)** Experimental design. **(B)** Representative data of the percentage of GP33 specific CD8^+^T cells (left panel), Histogram show the percentage and -fold expansion of GP33-specific cells in WT and WASp^−/−^ mice (middle and right panel). **(C)** The Lm load was calculated by serial dilution on BHI agar. Data are expressed as the mean ± SEM (*n* = 6 mice per group) and were analyzed using an unpaired *t*-test.

To evaluate the recall response of WASp^−/−^ antigen specific CD8^+^ T cells, we adoptively transferred equal numbers of GP33^+^CD8^+^ T cells (quantified by tetramer staining before adoptive transfer) from LCMV-infected WT or WASp^−/−^ mice (CD45.2) to congenic naïve recipients (CD45.1) on D150 PI; the recipients were challenged the next day with Lm-GP33 (Figure 8A). At 5 days post-challenge, we determined the -fold increase in the number of transferred GP33-specific CD8^+^ T cells by dividing the total number of GP33-specific CD8^+^ T cells recovered from the spleen by the number of GP33-specific CD8^+^ T cells transferred into individual naïve recipients before challenge. Compared with GP33-specific CD8^+^ memory T cells from WT mice, which increased 29.3-fold, cells from WASp^−/−^ mice increased by only 13.5-fold, indicating a marked reduction in expansion of WASp^−/−^ GP33-specific CD8^+^ cells (Figure 8B). Furthermore, Lm-GP33 titers in the livers of WASp^−/−^ mice were much higher than in those from WT mice (Figure 8C). Taken together, these results demonstrate that WASp is required for robust expansion of GP33-specific CD8^+^ memory T cells to generate protective recall responses.

### WASp Regulates CD8^+^ T Cell Memory Responses via a T Cell-Intrinsic Mechanism

**Figure 9:**
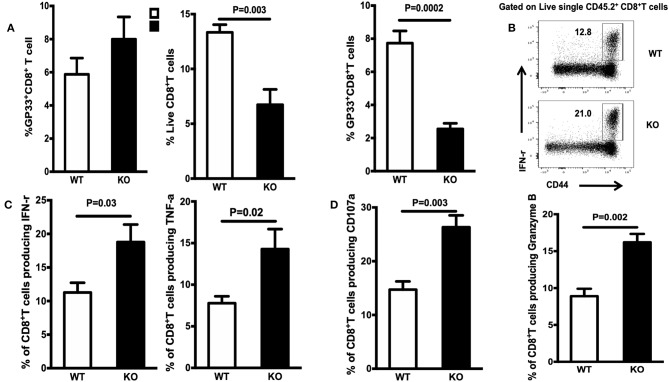
WASp regulates CD8^+^ T cell memory via T cell-intrinsic mechanisms. Bone marrow (BM) cells from WT (CD45.2) or WASp^−/−^ mice (CD45.2), and BM cells from WT (CD45.1) mice were mixed and adoptively transferred (intravenously at a 1:1 ratio) into lethally irradiated WT (CD45.1) mice. After reconstitution for at least 8 weeks, chimeric mice were infected with LCMV. Analysis was performed on Days 8 and 60 PI. All flow cytometry plots are gated on live CD45.2^+^CD8^+^ T cells (except A, middle panel, which was gated on live CD45.2^+^ cells). **(A)** Left panel: percentage of GP33-specific T cells within CD8 in chimeric mice on D8; middle and right panels, percentage of CD8 in lymphocytes and GP33-specific T cells within the CD8^+^ T cell population in chimeric mice on D60 PI. **(B)** Representative data showing the percentage of IFN-γ within CD8^+^T cells in chimeric mice on D60 PI. **(C)** Percentage of IFN-γ- or TNF-α-producing cells within CD8^+^T cells in chimeric mice on D60 PI. **(D)** Percentage of CD107α- or Granzyme B-producing T cells within CD8^+^T cells in chimeric mice on D60 PI. Data are expressed as the mean ± SEM (*n* = 5 mice per group) and were analyzed using an unpaired *t*-test.

To determine whether WASp plays a T cell-intrinsic role in regulating CD8^+^ T cell responses, we generated BM chimeras by reconstituting lethally irradiated CD45.1 mice with a mixture (1:1) of BM cells from WT/CD45.1 or WASp^−/−^/CD45.1 mice. After reconstitution for 8 weeks, recipient mice were infected i.p with LCMV-Armstrong (2 × 10^5^ PFU). Virus-specific CD8^+^ T cell responses were analyzed on Days 8 and 60 PI. Similar to the findings in WASp^−/−^ mice, the percentage of WASp^−/−^ GP33-specific CD8^+^ T cells in the spleen of chimeric mice was not significantly different from that of WT cells on Day 8 PI (Figure 9A, left panel); however, there was a substantial decrease in the percentage of WASp^−/−^ CD8^+^ T cells and GP33-specific CD8^+^ T cells in chimeric mice on Day 60 PI (Figure 9A, middle and right panels). Furthermore, the percentage of cytokine-producing WASp^−/−^ CD8^+^ T cells was consistently higher than that of WT CD8^+^ cells on D60 PI (Figures 9B–D). There was no difference in the CD45.1 compartment between chimeric mice from WT/CD45.1 and WASp^−/−^/CD45.1 BM (data not shown), which indicates that the environment in which WT and WASp^−/−^ BM cells develop is the same. These results demonstrate an intrinsic role for WASp in generating CD8^+^ memory T cells.

## Discussion

WASp plays a pivotal role in immune synapse formation, cytokine secretion, signal transduction, and migration and survival of T cells (7–10). Previous studies suggest a role for WASp in generating immune memory (19, 20). However, it is not clear whether WASp plays a T cell-intrinsic role in T cell response to virus, including cytokine production, effector-to-memory transition, and maintenance of functional CD8^+^ T cell memory.

Here, we systematically examined the role of WASp during expansion and contraction, and during memory and recall responses, of CD8^+^ T cell to acute viral infection. The results reveal that WASp acts in a CD8^+^ T cell-intrinsic manner to promote maintenance of CD8^+^ memory T cells, which is essential for generating protective recall response against re-infection by viruses. Impairment of CD8^+^ T cell memory responses may explain the increased susceptibility of WAS patients to recurrent viral infections as they age.

T cell priming requires interaction between T cells and antigen presenting cells (APCs), followed by signaling through immunological synapses. Aberrant T cell-APC interactions and signal transduction pathways are central dysfunctions that underlie the immune deficiencies reported in WAS patients (27). However, we found normal numbers of total CD8^+^ and GP-33 specific CD8^+^ cells during the expansion stage, suggesting that the interaction between WASp-deficient T cells and APCs is relatively intact, or even enhanced. One possible explanation for this finding is that the functional defects of WASp^−/−^ CD8^+^ T cells delay viral clearance; then, the increased viral load induces expansion of GP33-specific CD8^+^ T cells. Indeed, we found compromised clearance of virus from WASp^−/−^ mice on Day 8 PI, which is consistent with the results of a previous study showing reduced viral clearance from WASp^−/−^ mice (20).

CD8^+^ T cells from WAS patients show defective antigen-driven proliferation and cytokine production *in vitro*; WASp^−/−^ CD8^+^ T cells show profound impairment of IL-2, IFN-γ, and TNF-α production due to defective gene transcription (9, 11). However, CD8^+^ cells from WAS patients show intact secretion of perforin and Granzyme B (12). Surprisingly, we found an increase in the percentage of cytokine-producing CD8^+^ T cells in response to cognate antigen stimulation, even when the percentage of antigen-specific CD8^+^ T cells decreased. This increase cannot be explained only by delayed clearance of virus because even when the virus was eliminated completely from WT and WASp^−/−^ mice on Day 30 PI, the percentage of cytokine-producing CD8^+^ T cells in WASp^−/−^ mice remained higher. This is contrast to the results of previous studies (20, 28). However, it should be noted that the experiments in the previous study were done on D6 and D7 PI, at a time when the number of antigen-specific CD8^+^ T cells had not reached a peak. These results are in accordance with those of another study (29) in which the experiments were done on D50 PI. Also, they are in line with our finding that the proportion of TCM within the GP33-specific cell population in WT mice increased (TCMs have little or no effector function) (30). This may contribute to the reduced proportion of the cytokine-producing CD8^+^T cells in WT mice. As chronic exposure to IFN-γ can trigger autoimmune diseases (31), cytokine-producing CD8^+^ T cells are potentially autoreactive, although this hypothesis remains to be tested.

T lymphocytes from the peripheral blood of WAS patients exhibit accelerated apoptosis, while at the same time displaying a marked increase in CD95 expression and attenuated expression of Bcl-2 (6, 7). Our previous study, and report by other group, show that the numbers of naïve T cells in WAS patients are reduced markedly (5, 32). This reduction may be the reason for attenuated expression of BCL-2 and increased expression of CD95. Here, we found an increase in apoptosis and a decrease in the numbers of live GP33-specific CD8^+^ T cells in WASp^−/−^ mice after antigenic stimulation. The increase in apoptosis in WASp^−/−^ mice was associated with high expression of CD95 and FASL by GP33-specific CD8^+^ cells. However, expression of BCL-2 by GP33-specific CD8^+^ T cells from WASp^−/−^ mice was equivalent to that in WT mice. These results indicate that increased pro-apoptotic signals, rather than anti-apoptotic signals, contribute to the reduction in antigen-specific CD8^+^ memory T cells in WASp^−/−^ mice.

Memory CD8^+^ T cells can be identified by their capacity to mount rapid and protective recall responses (33). Previous work identified IL-7R as a marker of memory precursor effector cells following acute infection (34). A reduction in the number of IL-7R^+^ GP33-specific cells was detected in WASp^−/−^ mice on Days 15 and Day 30, which suggests that fewer memory cells are generated in WASp^−/−^ mice at later time points. Indeed, the percentage of GP33-specific CD8^+^ T cells in WASp^−/−^ mice decreased markedly on Day 60 and later, and the absolute number of GP33-specific CD8^+^ T cells decreased on Days 200 and 255, suggesting that IL-7R is a useful marker for memory precursor cells. However, it should be noted that the percentage of IL-7R positive GP33 specific T cell is even decreased on D60 compared with that on D30, the reasons for this dynamic regulation of IL-7R are not clear but suggest that there might be a period of time during infection when T cells are refractory to further upregulation of IL-7R.

Viral or bacterial infection can induce generation of two subpopulations of antigen-specific memory CD8^+^ T cells (35): TCM and TEM. TCMs have a much higher capacity to reconstitute the memory T cell pool than the effector pool (36). We determined the percentage of TCM within the GP33-specific CD8^+^ T cell population using CD62L as a surface marker. We found that the percentage of CD62L-expressing TCM within the GP33-specific CD8^+^T cell population in WASp^−/−^ mice decreased on Day 15 and later, which is consistent with reduced production of GP33-specific memory T cells at later time points. These data suggest that although the quantity of GP33-specific CD8^+^ T cells in WASp^−/−^ mice is unchanged during the contraction and early memory stages, they do not differentiate efficiently into TCM.

CD8^+^ T cells can mount efficient antiviral responses. However, if the protection is not sufficiently robust, a chronic viral infection can develop. Under such conditions, T cell exhaustion can occur (37). Indeed, we found that the percentage of exhausted GP33-specific CD8^+^ T cells was higher in WASp^−/−^ mice than in WT mice. This is in line with the delayed viral clearance observed in WASp^−/−^ mice. However, our data also show that the number of GP33-specific CD8^+^T cells in WASp^−/−^ mice was not altered until D200 PI, and that expression of CD107a and Granzyme B production by these T cells increased; this is contrast to the delayed viral clearance observed in WASp^−/−^ mice. However, it can be explained by the fact that granule polarization in CD8^+^ T cells is incomplete (12) Functional impairment of other immune cells, including NK cells (38) and DCs (20), may also contribute the delayed viral clearance observed in WASp^−/−^ mice.

Immunological memory is the hallmark of acquired immunity. Memory lymphocytes confer immediate protection on peripheral tissues and mount rapid and robust recall responses to antigens in secondary lymphoid organs. WASp^−/−^ mice mount much weaker recall responses to influenza A than WT mice (19). It should be noted that in this elegant study, the number of antigen-specific CD8^+^ memory T cells detected in WASp^−/−^ mice during the initial response to influenza was also reduced. Using an adoptive transfer approach, our study demonstrates a critical role for WASp in CD8^+^ T recall responses and protective immunity against viral re-infection.

It is not clear whether WASp regulates formation of memory CD8^+^ T cells directly via cell-intrinsic mechanisms, or indirectly through other immune cells. CD4^+^ cells and DCs are required for generation of memory CD8^+^ T cells (39, 40). DCs are required for activation of memory CD8^+^ T cells and a subsequent robust recall response (40). The number and function of DCs and CD4^+^ cells in WASp^−/−^ mice are reduced (11, 17, 18), which potentially impairs memory CD8^+^ T cells. Here, we used BM chimera mice to provide evidence for a CD8^+^ cell-intrinsic role for WASp in maintaining LCMV-specific memory CD8^+^ T cells.

In summary, WASp acts as a key regulator of CD8^+^ T cell memory by promoting CD8^+^ effector-to-memory conversion, survival of CD8^+^ memory T cells, and recall responses. Our findings increase our understanding of the mechanisms underlying the homeostasis of CD8^+^ memory T cells and have potential implications for development of new vaccine strategies that can increase CD8^+^ T cell memory responses by modulating the activity of WASp.

## Data Availability Statement

The raw data supporting the conclusions of this manuscript will be made available by the authors, without undue reservation, to any qualified researcher.

## Ethics Statement

The animal study was reviewed and approved by the Institutional Animal Care and Use Committees of Chong Qing Medical University.

## Author Contributions

XZ designed the experiments. WS and XZ supervised the experiments. QL, LZha, ZS, TY, and LZho performed the experiments. QL analyzed the data. QL, WS, and XZ wrote the paper.

### Conflict of Interest

The authors declare that the research was conducted in the absence of any commercial or financial relationships that could be construed as a potential conflict of interest.

## Supplementary Material

Supplemental Figure 1 CD8^+^ T cells from uninfected WASp^−/−^ mice show an altered phonotype. Mice were sacrificed at 6–8 weeks-of-age and the phonotype of CD8^+^ T cells in the spleen was determined by flow cytometry. **(A)** Percentage and absolute number of CD8^+^ T cells in spleen of WT and WASp^−/−^ mice. **(B)** Percentage of naïve T cells within the CD8^+^ T cell population and absolute number of naive CD8^+^ T cells in spleen of WT and WASp^−/−^ mice. **(C)** Percentage of TCM within the CD8^+^ T cell population and absolute number of TCM CD8^+^ T cells. **(D)** Percentage of TEM within the CD8^+^ T cell population and absolute number of TEM CD8^+^ T cells in spleen of WT and WASp^−/−^ mice. Data are expressed as the mean ± SEM and are representative of at least two independent experiments, each with *n* = 5 mice per group. Data were analyzed using an unpaired *t*-test.

Supplemental Figure 2 The phonotype of GP33 specific CD8^+^ T cells is unaltered, but viral clearance is delayed, in WASp^−/−^ mice during the expansion stage. WT and WASp^−/−^ mice were sacrificed at 8 days PI. Splenocytes were isolated and stained for CD8, D^b^/GP33 tetramer, CD44, CD62L, and then stained intracellularly for Ki67, T-bet, and BCL-2. **(A)** Expression of BCL-2, Ki 67, and T-bet within GP33-specific CD8^+^ T cells (*n* = 4 mice per group). **(B)** Viral titer was determined by QPCR (*n* = 5 mice per group). Data are expressed as the mean ±SEM and analyzed using an unpaired *t*-test.

Supplemental Figure 3 WASp does not affect development of CD8^+^ T cells in the thymus. Uninfected WASp^−/−^ and WT mice aged 6–8 weeks were sacrificed and development of CD8^+^ T cells was analyzed by flow cytometry. **(A)** Representative flow cytometry plots showing the percentage of CD8 within thymocytes (left panel), CD69, Qa-2 expression within CD8^+^T cells (middle panel), and CD62L expression within CD8^+^T cells by WT and WASp^−/−^ thymocytes (right panel). **(B)** Graph showing the percentage of CD8^+^T cells within thymocytes (left panel), CD69^+^ Qa-2^+^ (middle panel), and CD62L^+^ cells (right panel) within CD8^+^T cells in the WT and WASp^−/−^ thymocytes. Data are expressed as the mean ± SEM and are representative of at least two independent experiments, each with *n* = 5 mice per group. Data were analyzed using an unpaired *t*-test.
