# Supplementary figures and images for: WASp Is Essential for Effector-to-Memory conversion and for Maintenance of CD8+T Cell Memory
Source: Front Immunol. 2019 Sep 24;10:2262. doi: 10.3389/fimmu.2019.02262 (PMC6769127; doi:10.3389/fimmu.2019.02262)

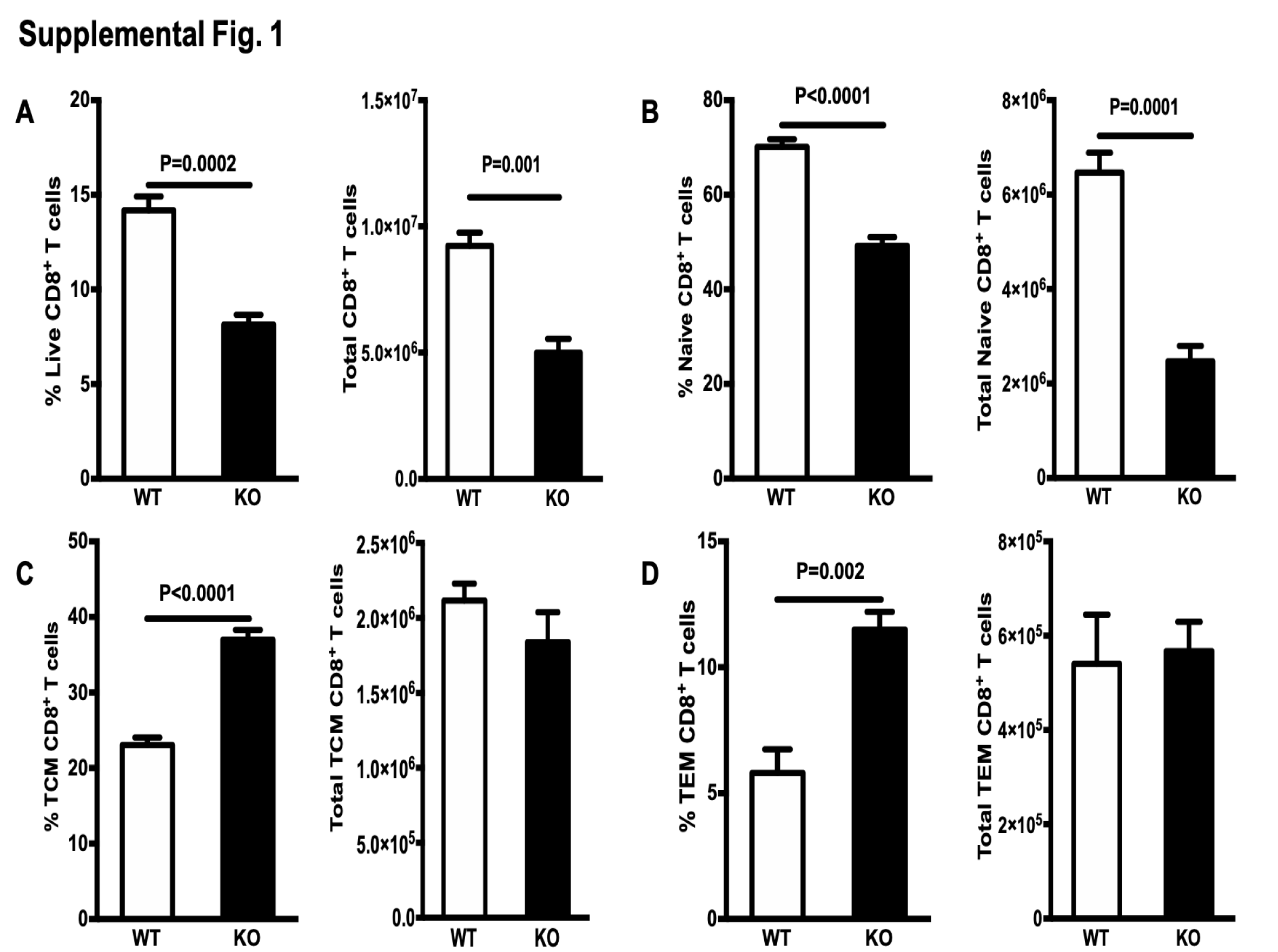

Supplement: Supplemental Figure 1 — CD8+ T cells from uninfected WASp−/− mice show an altered phonotype. Mice were sacrificed at 6–8 weeks-of-age and the phonotype of CD8+ T cells in the spleen was determined by flow cytometry. (A) Percentage and absolute number of CD8+ T cells in spleen of WT and WASp−/− mice. (B) Percentage of naïve T cells within the CD8+ T cell population and absolute number of naive CD8+ T cells in spleen of WT and WASp−/− mice. (C) Percentage of TCM within the CD8+ T cell population and absolute number of TCM CD8+ T cells. (D) Percentage of TEM within the CD8+ T cell population and absolute number of TEM CD8+ T cells in spleen of WT and WASp−/− mice. Data are expressed as the mean ± SEM and are representative of at least two independent experiments, each with n = 5 mice per group. Data were analyzed using an unpaired t-test. [file Image_1.TIF]

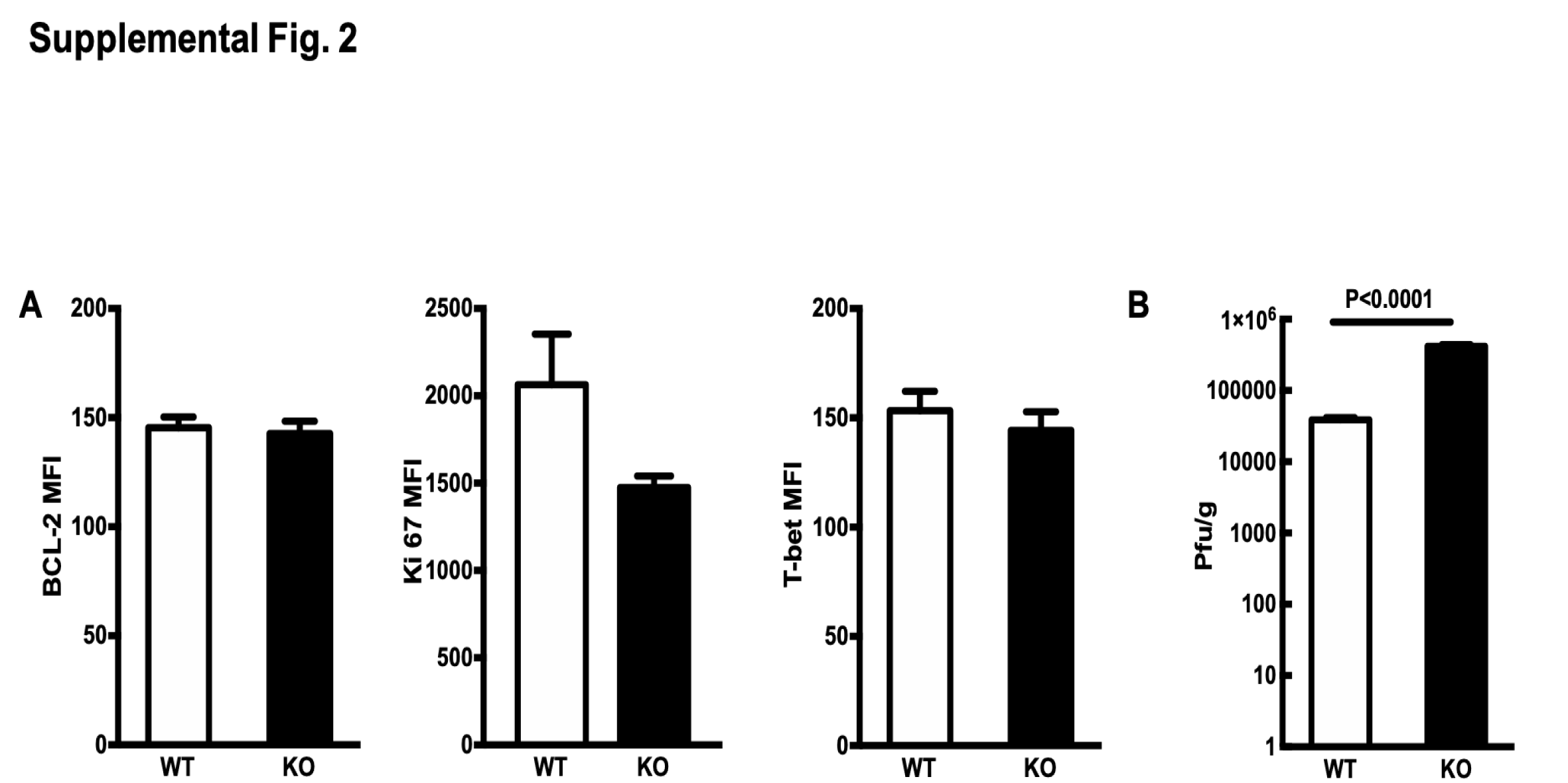

Supplement: Supplemental Figure 2 — The phonotype of GP33 specific CD8+ T cells is unaltered, but viral clearance is delayed, in WASp−/− mice during the expansion stage. WT and WASp−/− mice were sacrificed at 8 days PI. Splenocytes were isolated and stained for CD8, Db/GP33 tetramer, CD44, CD62L, and then stained intracellularly for Ki67, T-bet, and BCL-2. (A) Expression of BCL-2, Ki 67, and T-bet within GP33-specific CD8+ T cells (n = 4 mice per group). (B) Viral titer was determined by QPCR (n = 5 mice per group). Data are expressed as the mean ±SEM and analyzed using an unpaired t-test. [file Image_2.TIF]

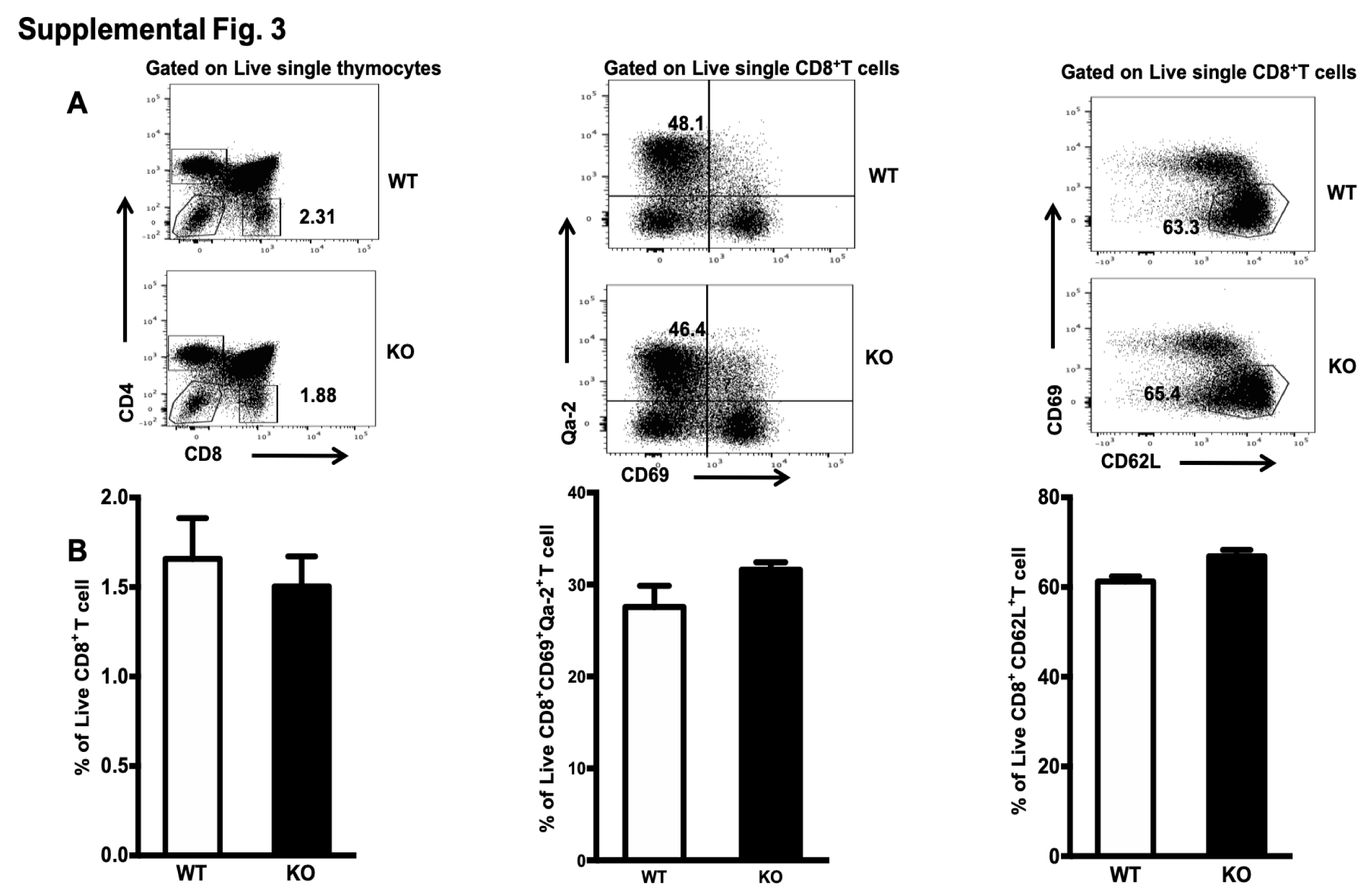

Supplement: Supplemental Figure 3 — WASp does not affect development of CD8+ T cells in the thymus. Uninfected WASp−/− and WT mice aged 6–8 weeks were sacrificed and development of CD8+ T cells was analyzed by flow cytometry. (A) Representative flow cytometry plots showing the percentage of CD8 within thymocytes (left panel), CD69, Qa-2 expression within CD8+T cells (middle panel), and CD62L expression within CD8+T cells by WT and WASp−/− thymocytes (right panel). (B) Graph showing the percentage of CD8+T cells within thymocytes (left panel), CD69+ Qa-2+ (middle panel), and CD62L+ cells (right panel) within CD8+T cells in the WT and WASp−/− thymocytes. Data are expressed as the mean ± SEM and are representative of at least two independent experiments, each with n = 5 mice per group. Data were analyzed using an unpaired t-test. [file Image_3.TIF]
